# Supplementary material for: Extramacrochaetae regulates Notch signaling in the Drosophila eye through non-apoptotic caspase activity
Source: bioRxiv. 2024 Jul 29:2023.10.04.560841. Preprint. [Version 2] doi: 10.1101/2023.10.04.560841 (PMC11312471; doi:10.1101/2023.10.04.560841)
Supplement: Supplementary file 1 [file NIHPP2023.10.04.560841v2-supplement-1.pdf]

bioRxiv preprint doi: https://doi.org/10.1101/2023.10.04.560841; this version posted July 29, 2024. The copyright holder for this preprint (which was not certified by peer review) is the author/funder, who has granted bioRxiv a license to display the preprint in perpetuity. It is made available under aCC-BY 4.0 International license.

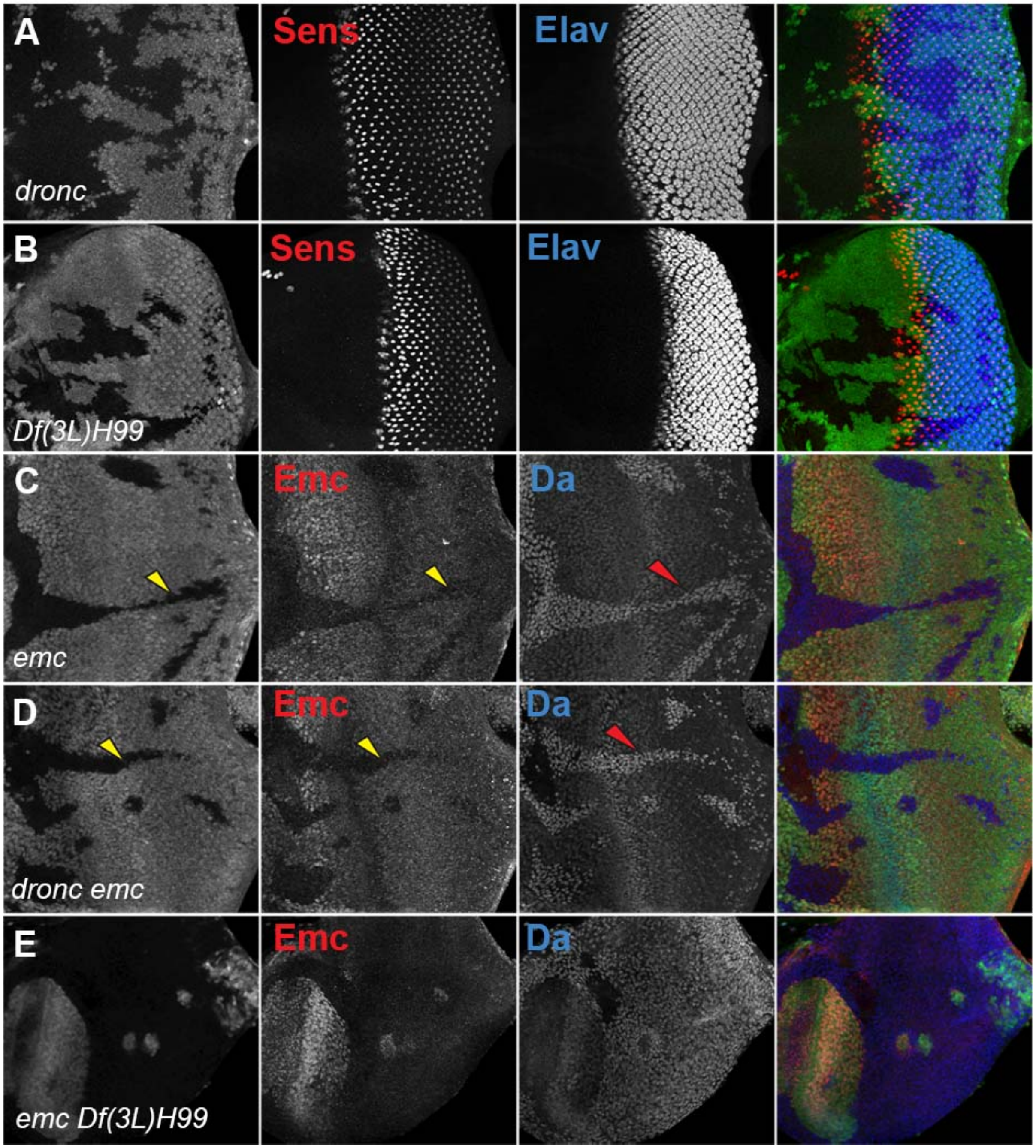

Figure 2 Figure Supplement 1: Morphogenetic furrow progression in *emc* mutant cells
In eye imaginal discs, normal furrow progression was observed in (A) *dronc* $^{-/-}$ and (B) *Df(3L)H99* homozygous clones, indicated by Senseless and Elav staining. Homozygous mutant clones of *emc*$^{-/-}$ (C) lacking GFP expression, have no Emc staining (yellow arrowhead) and higher Da (red arrowhead). At the morphogenetic furrow as shown by yellow arrow, Emc expression goes down and Da goes up. Similar results were observed in (D) *dronc*$^{-/-}$ *emc*$^{-/-}$ clones and (E) *emc*$^{-/-}$ *Df(3L) H99* $^{-/-}$ clones confirm that these are *emc* null clones. Genotypes: (A) *ywhsF;;dronc*^{i29}*FRT80/[UbiGFP] M(3)67C FRT80* (B) *ywhsF;; Df(3L)H99 FRT80/[UbiGFP] M(3)67C FRT80*. (C) *ywhsF;;emc*^{AP6}*FRT80/[Ubi-GFP] M(3)67C FRT80* (D)

bioRxiv preprint doi: https://doi.org/10.1101/2023.10.04.560841; this version posted July 29, 2024. The copyright holder for this preprint (which was not certified by peer review) is the author/funder, who has granted bioRxiv a license to display the preprint in perpetuity. It is made available under aCC-BY 4.0 International license.

ywhsF;;dronc^{i29}emc^{AP6} FRT80/[UbiGFP] M(3)67C FRT80 (E) *ywhsF;;emc^{AP6} Df(3L)H99 FRT80/[UbiGFP] M(3)67C FRT80*. N=4 for each genotype.

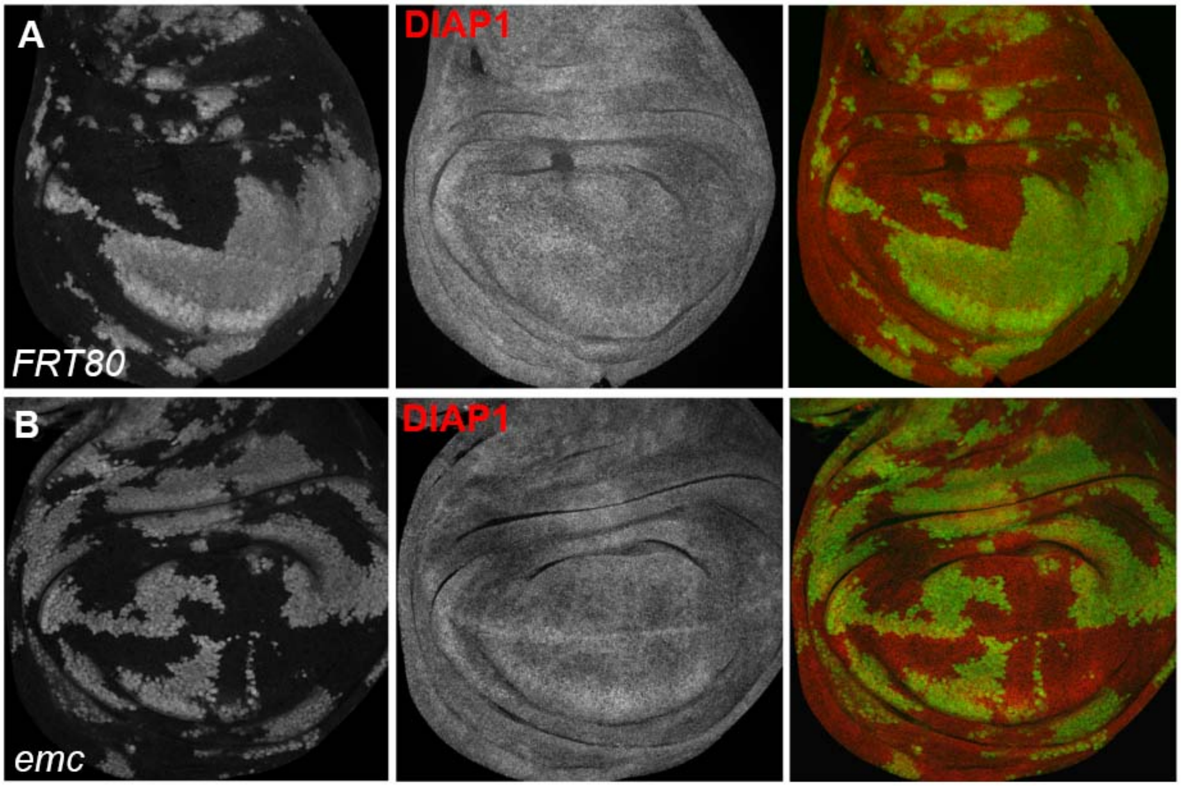

Figure 3 Figure Supplement 1: DIAP1 staining in wing disc clones. In wing discs of emc clones (B) we did not detect changes in Diap1 levels in comparison to control clones (A). Genotypes: (A) *ywhsF;;FRT80/[UbiGFP] M(3)67C FRT80* (B) *ywhsF;;emc^{AP6}FRT80/[Ubi-GFP] M(3)67C FRT80.*

bioRxiv preprint doi: https://doi.org/10.1101/2023.10.04.560841; this version posted July 29, 2024. The copyright holder for this preprint (which was not certified by peer review) is the author/funder, who has granted bioRxiv a license to display the preprint in perpetuity. It is made available under aCC-BY 4.0 International license.

Figure 3 Supplement 2

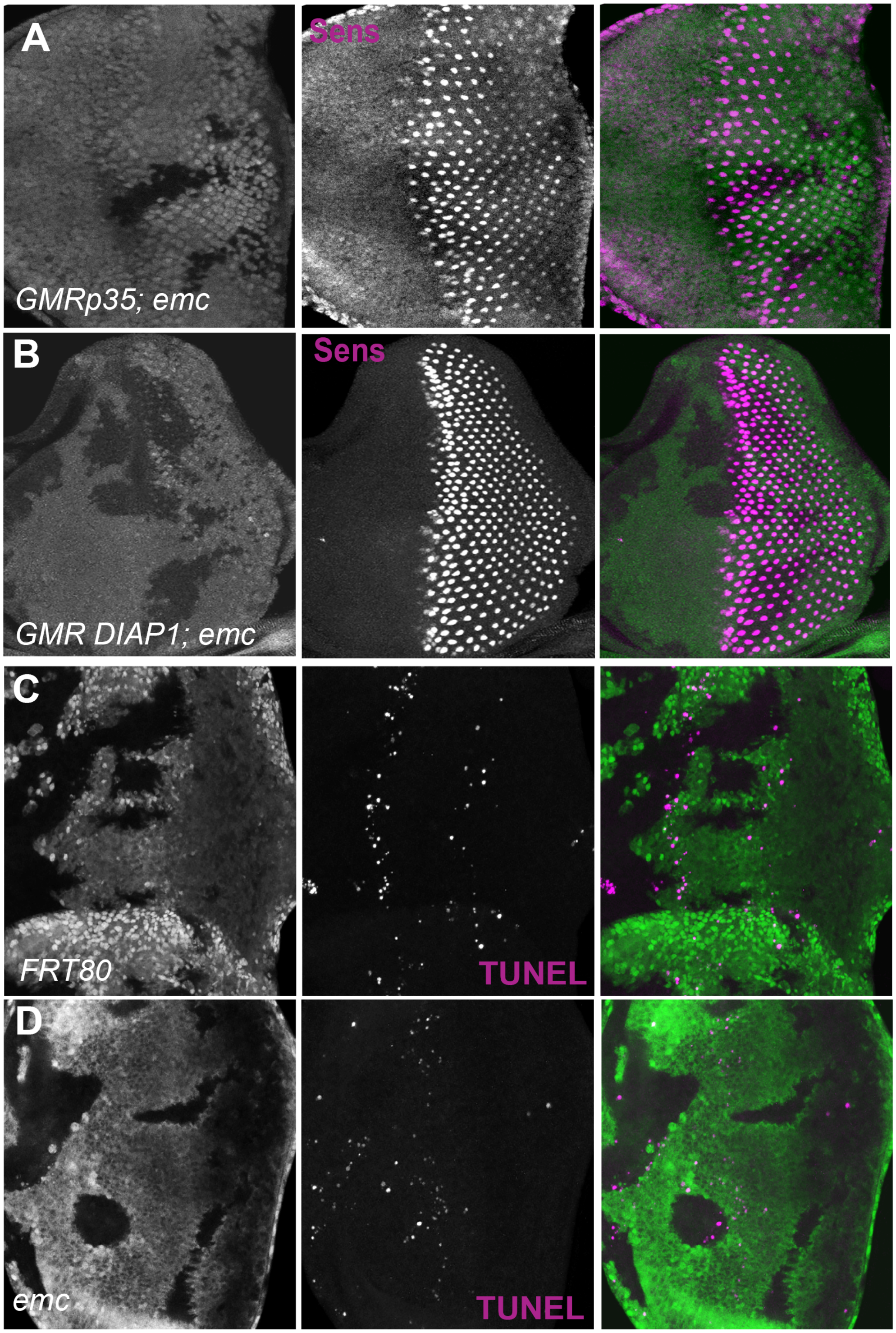

733
734

bioRxiv preprint doi: https://doi.org/10.1101/2023.10.04.560841; this version posted July 29, 2024. The copyright holder for this preprint (which was not certified by peer review) is the author/funder, who has granted bioRxiv a license to display the preprint in perpetuity. It is made available under aCC-BY 4.0 International license.

Figure 3 Figure Supplement 2: Apoptosis and furrow progression Representative images of eye imaginal discs stained for senseless. (A) In GMR-DIAP1 eye disc mutant for emc furrow progression is normal and is marked by Senseless staining. Similarly, in GMR-p35 eye disc mutant for emc also show normal furrow progression (B). Representative images of eye imaginal discs with TUNEL staining. (A) ywhsF/GMR-DIAP1;;*emc*^{AP6}*FRT80/[Ubi-GFP] M(3)67C FRT80* B) ywhsF/GMR-p35;;*emc*^{AP6}*FRT80/[Ubi-GFP] M(3)67C FRT80*. (C) *ywhsF;;FRT80/[UbiGFP] M(3)67C FRT80* (D) *ywhsF;;emc*^{AP6}*FRT80/[Ubi-GFP] M(3)67C FRT80*. N=4 for each genotype.

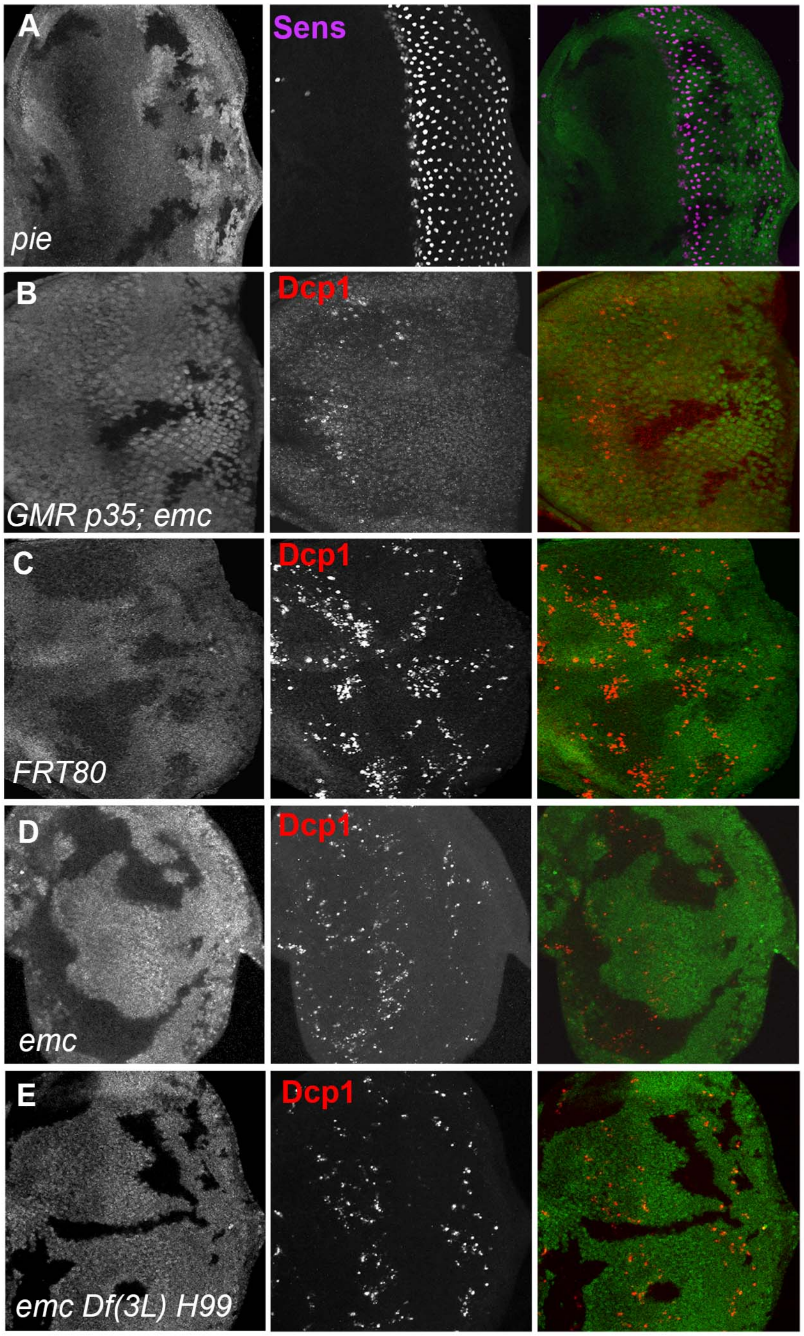

bioRxiv preprint doi: https://doi.org/10.1101/2023.10.04.560841; this version posted July 29, 2024. The copyright holder for this preprint (which was not certified by peer review) is the author/funder, who has granted bioRxiv a license to display the preprint in perpetuity. It is made available under aCC-BY 4.0 International license.

Figure 3 Figure Supplement 3: Emc clones show no caspase activity beyond the morphogenetic furrow. In *pie*^{EB3} homozygous clones, furrow progression occurs at normal speed marked by Senseless staining in R8 photoreceptors (A). In GMR-p35 eye disc mutant for emc show lack of Dcp1staining (B). In *emc* mutant clones posterior to morphogenetic furrow show lack of Dcp1 staining (D) in comparison to FRT80 clones (C). Genotypes: (A) ywhsF/+; *pie*^{EB3}*FRT40/FRT40Alz*. **(B)** ywhsF/GMR-p35;;*emc*^{AP6}*FRT80/[Ubi-GFP] M(3)67C FRT80* (C) *ywhsF;;FRT80/[UbiGFP] M(3)67C FRT80* (D) *ywhsF;;emc*^{AP6}*FRT80/[Ubi-GFP] M(3)67C FRT80*. (E) *ywhsF;;emc*^{AP6} *Df(3L)H99 FRT80/[UbiGFP] M(3)67C FRT80*. N=4 for each genotype.

Figure 4 figure supplement 1

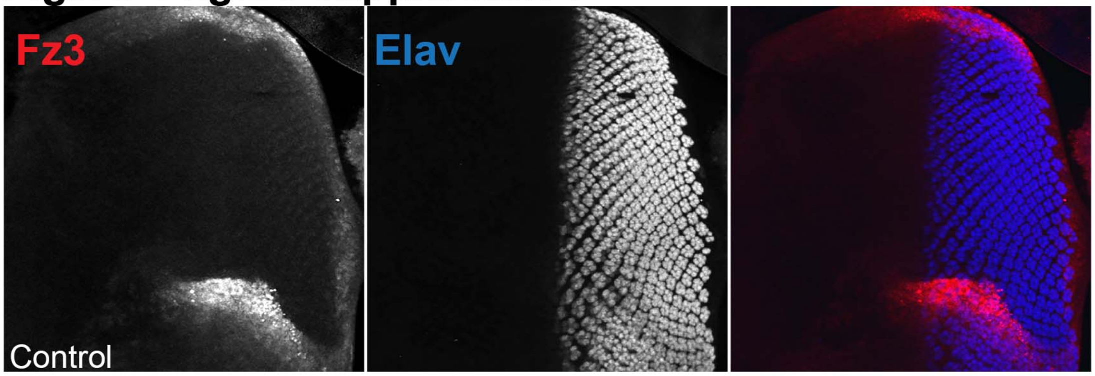

Figure 4 Figure Supplement 1: Fz3-RFP/+ eye disc showing RFP and Elav staining. N=3.

Figure 5 figure supplement 1

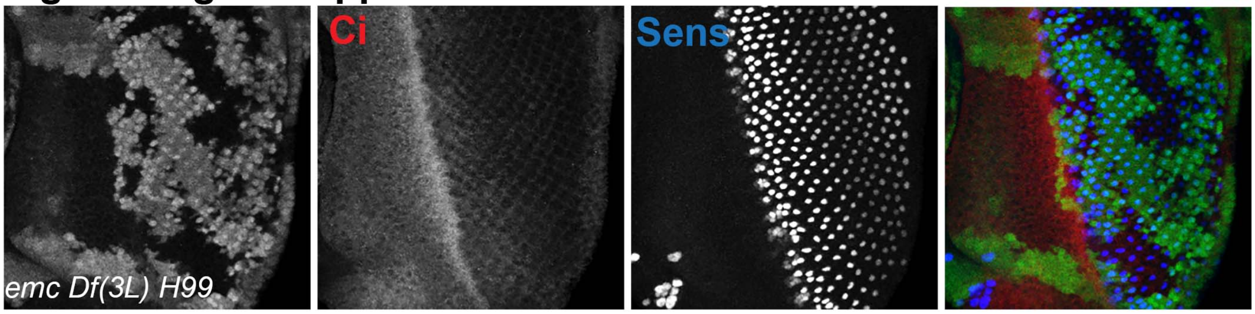

Figure 5 Figure Supplement 1: Ci155 levels when cell death pathways are blocked Representative image of eye imaginal disc in *emc H99* clones in the non-Minute background, showing similar areas inside and outside clones. Genotype: *ywhsF;; emc*^{AP6} *Df(3L)H99 FRT80/[UbiGFP]FRT80*. N=4.

bioRxiv preprint doi: https://doi.org/10.1101/2023.10.04.560841; this version posted July 29, 2024. The copyright holder for this preprint (which was not certified by peer review) is the author/funder, who has granted bioRxiv a license to display the preprint in perpetuity. It is made available under aCC-BY 4.0 International license.

Figure 5 Figure Supplement 2

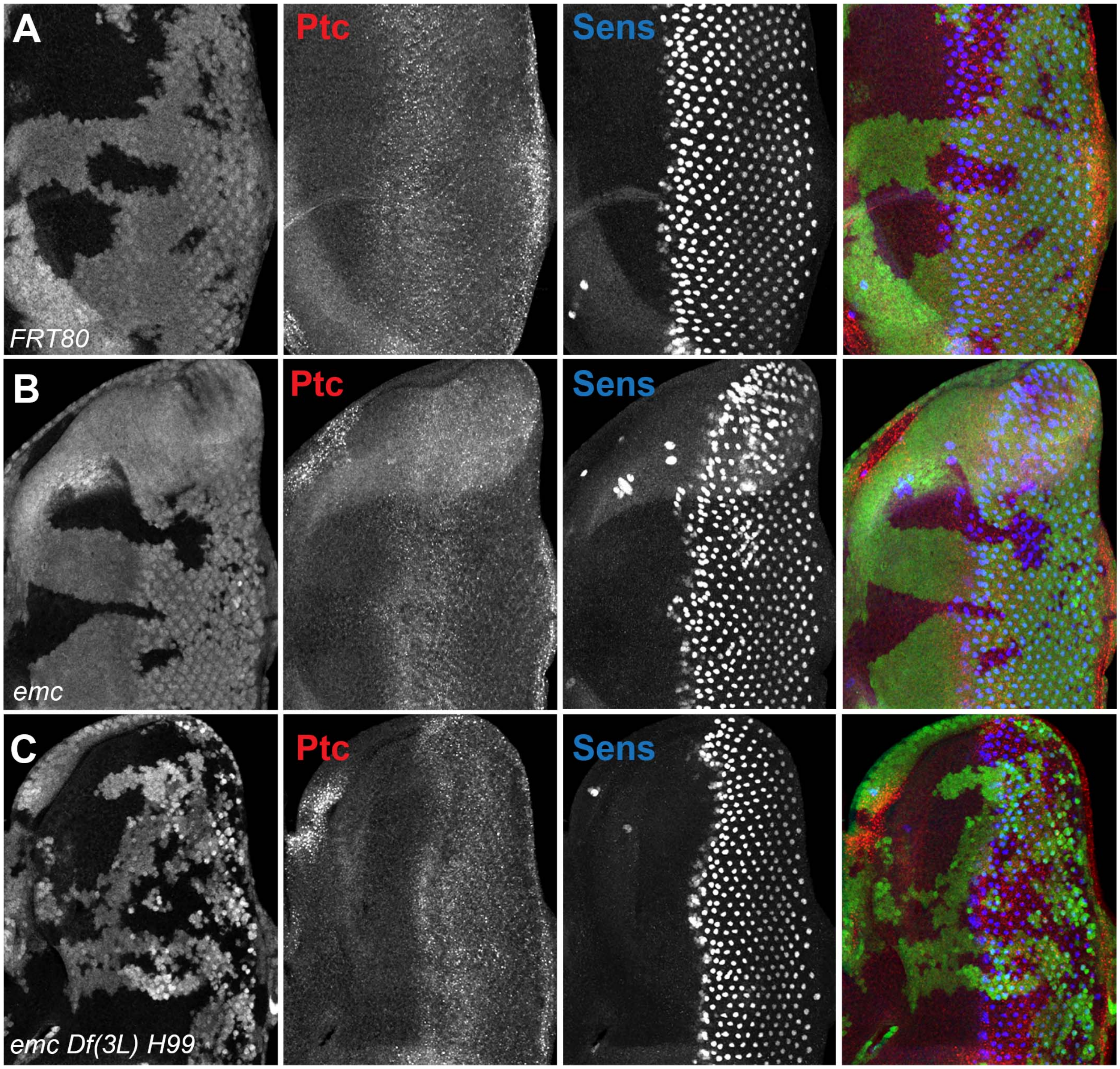

Figure 5 Figure Supplement 2: Patched staining in emc mutants

(A) *ywhsF;;FRT80/[UbiGFP] M(3)67C FRT80* (B) *ywhsF;;emc^{AP6}FRT80/[Ubi-GFP] M(3)67C FRT80* (C) *ywhsF;;emc^{AP6} Df(3L)H99 FRT80/[UbiGFP] M(3)67C FRT8.*

bioRxiv preprint doi: https://doi.org/10.1101/2023.10.04.560841; this version posted July 29, 2024. The copyright holder for this preprint (which was not certified by peer review) is the author/funder, who has granted bioRxiv a license to display the preprint in perpetuity. It is made available under aCC-BY 4.0 International license.

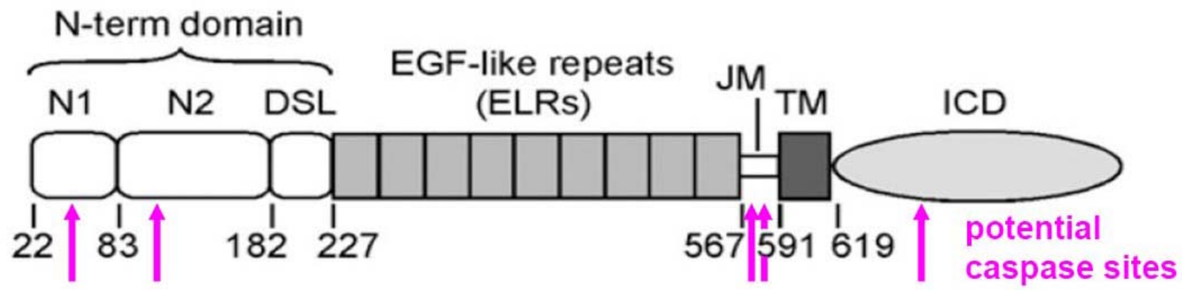

Figure 7 Figure Supplement 1: Potential Caspase sites predicted in the Delta protein._ Like many proteins, Delta has multiple potential caspase cleavage sites, here predicted suing Cascleave 2.0 (Wang et al., 2014). None are high-confidence predictions and only one is in the intracellular domain where caspase access is plausible (position 675, predicted score 0.647). The intracellular domain is required for Delta signaling. Cleavage at position 675 would remove the main ubiquitylation site required for signaling, as well as the binding site for *mindbomb*, so is not anticipated to enhance signaling activity (Daskalaki et al., 2011).
